# A hands-on introduction to querying evolutionary relationships across multiple data sources using SPARQL

**DOI:** 10.12688/f1000research.21027.2

**Published:** 2020-07-22

**Authors:** Ana Claudia Sima, Christophe Dessimoz, Kurt Stockinger, Monique Zahn-Zabal, Tarcisio Mendes de Farias

**Affiliations:** 1 ZHAW Zurich University of Applied Sciences, Winterthur, Zurich, Switzerland; 2 Department of Computational Biology, University of Lausanne, Lausanne, Vaud, Switzerland; 3 SIB Swiss Institute of Bioinformatics, Lausanne, Vaud, Switzerland; 4 Center for Integrative Genomics, University of Lausanne, Lausanne, Vaud, Switzerland; 5 Department of Computer Science, University College London, London, UK; 6 Department of Genetics, Evolution, and Environment, University College London, London, UK; 7 Department of Ecology and Evolution, University of Lausanne, Lausanne, Vaud, Switzerland

**Keywords:** Orthology, Comparative Genomics, Sequence Homology, Resource Description Framework (RDF), SPARQL

## Abstract

The increasing use of Semantic Web technologies in the life sciences, in particular the use of the Resource Description Framework (RDF) and the RDF query language SPARQL, opens the path for novel integrative analyses, combining information from multiple data sources. However, analyzing evolutionary data in RDF is not trivial, due to the steep learning curve required to understand both the data models adopted by different RDF data sources, as well as the equivalent SPARQL constructs required to benefit from this data – in particular, recursive property paths. In this article, we provide a hands-on introduction to querying evolutionary data across several data sources that publish orthology information in RDF, namely: The Orthologous MAtrix (OMA), the European Bioinformatics Institute (EBI) RDF platform, the Database of Orthologous Groups (OrthoDB) and the Microbial Genome Database (MBGD). We present four protocols in increasing order of complexity. In these protocols, we demonstrate through SPARQL queries how to retrieve pairwise orthologs, homologous groups, and hierarchical orthologous groups. Finally, we show how orthology information in different data sources can be compared, through the use of federated SPARQL queries.

## Introduction

Gene classification based on evolutionary history is essential for many aspects of comparative and functional genomics - reviewed in ( Gabaldón & Koonin, 2013); ( Glover *et al.*, 2019). On the one hand, evolutionary relations are often described as binary relations. Two genes that share a common ancestor are defined as *homologs*. We can classify homologs into *orthologs*, which originate from a speciation event; *paralogs*, which originate from a gene duplication; and *xenologs*, which originate from horizontal gene transfer ( Fitch, 1970). On the other hand, Hierarchical Orthologous Groups (HOGs) are hierarchical clusters of corresponding genes where each level in the hierarchy refers to a common ancestral gene at a taxonomic level of reference ( Altenhoff *et al.*, 2013). Further details about orthology, paralogy, xenology and various kinds of groupwise orthology relationships are described in ( Fernández *et al.,* 2020). Identifying orthologs and HOGs is valuable in several contexts such as gene function inference, gene evolution dynamics and comparative genomics.

To query and interoperate biological databases, Semantic Web Technologies are being increasingly adopted, in particular the use of the Resource Description Framework (RDF) and SPARQL protocol and RDF query language ( SPARQL Query Language for RDF, n.d.). However, despite the progress they have enabled in several fields, particularly in the life sciences ( Duek *et al.*, 2018), ( Iyappan *et al.*, 2016) ( Williams *et al.*, 2012), there are still significant challenges that limit their use for the larger scientific community. In particular, analysing evolutionary relationship data in RDF poses the following challenges:

1) *complex* data models - for example, while storing data in a hierarchical structure (HOGs) results in significant performance benefits for common analyses, such as computing orthologs of a specific gene in a different model organism, the hierarchy also results in requiring advanced knowledge of the SPARQL language (in particular, recursivity) in order to benefit from the RDF representation of HOGs. In this article, we present a series of hands-on examples, in increasing order of complexity, to familiarise the reader with the basic concepts needed to query evolutionary relationships in orthology databases.

2) *heterogeneous* data models - understanding the data model of a *single* orthology database might not be sufficient in general, since different databases have made different design decisions. We help overcome this challenge by depicting how the following major Orthology Databases structure their data in RDF, as well as how they can be queried using SPARQL: the Orthologous MAtrix (OMA) ( Altenhoff *et al.*, 2018), the European Bioinformatics Institute (EBI) RDF platform ( Jupp *et al.*, 2014), the Database of Orthologous Groups (OrthoDB) ( Zdobnov *et al.*, 2017) and the Microbial Genome Database (MBGD) ( Uchiyama *et al.*, 2018).

3) *overhead of integration* into existing analysis pipelines. The limited rate of adoption of Semantic Web Technologies can be explained by the reluctance of bioinformaticians to change their existing workflows in order to accommodate new data formats based on the RDF framework. For example, retrieving orthology information using public SPARQL endpoints instead of the more traditional file-based data exchange or full database dumps. A SPARQL endpoint is an access point for receiving and processing SPARQL protocol requests. In this article, we show through concrete examples that integrating the results of SPARQL queries into existing analyses is a straightforward task - more specifically, we show how to transform the results into regular Pandas dataframes in Python. Furthermore, we provide an accompanying Jupyter notebook where all the examples presented in this article can be directly tested and further refined.

This article has several goals:

(1) *Understanding Orthology Data Models.* Become familiar with how evolutionary relationships are represented in RDF across several databases. Learn about the data modelling decisions: common points as well as differences between these data sources to support the choice of one or more of them for a given analysis.
(2) *Understanding how to query orthology data using SPARQL.* To this end, we extend the introduction and examples in ( Chiba *et al.*, 2015) ( de Farias *et al.*, 2017), while also covering multiple, distributed orthology data sources.
(3) *Integrating external sources.* Leverage connections to other external bioinformatics resources that make their data available in public SPARQL endpoints based on cross-references. In particular, learn about the role of UniProt cross-references as a *bridge* between different data sources in integrative analyses.

In addition, we show how to use SPARQL to make meta-analyses combining multiple orthology databases. For instance, for a given gene, which are the orthologs in a given database which are *not* present in another one? Finally, we show how to leverage SPARQL aggregations in order to get useful statistics about orthology data available in the sources.

Finally, learn how to leverage SPARQL results in downstream analyses by converting them to Pandas dataframes. This is illustrated through a series of hands-on exercises in the accompanying Jupyter notebook (exercises provided in Python).

The protocols presented in this article are aimed at bioinformaticians who are already familiar with the basics of SPARQL and wish to learn how orthology data can be integrated in their research analyses programmatically, through the use of (federated) SPARQL queries.

## Materials

In the following paragraphs, we briefly describe the orthology databases considered in this article.

**OrthoDB** ( Zdobnov *et al.*, 2017) contains orthologous genes along with evolutionary and functional annotations. It relies on HOGs to enable different orthology information resolutions with regards to more closely related species. The 2018 OrthoDB version covers thousands of eukaryotes, prokaryotes, and viruses. OrthoDB data is available in RDF through the public SPARQL endpoint at https://sparql.orthodb.org/sparql. We note here that the timeout for the public SPARQL endpoint is limited to 100 seconds - more precisely, queries with longer *estimated* execution time will *not* be allowed to run.

**MBGD** ( Uchiyama *et al.*, 2018) is a comparative genomics database that contains orthology information about bacteria, archaea and unicellular eukaryotes. The 2018 MBGD version has more than six thousand genomes. The MBGD SPARQL endpoint is available online at http://mbgd.genome.ad.jp/sparql/.

**OMA** ( Altenhoff *et al.*, 2018) provides orthologous gene inferences covering all three domains of life: Archaea, Bacteria, and Eukarya. Although mainly focusing on orthology information, OMA also provides paralogy information (i.e. genes related by duplication). Other homology information is not explicitly available but might be manually or automatically extracted from HOGs ( de Farias *et al.*, 2017). The 2020 OMA version has 2326 species and can be queried through the SPARQL endpoint at https://sparql.omabrowser.org/lode/sparql. OMA reports multiple kinds of pairwise and groupwise orthologous relationships, described in ( Zahn-Zabal *et al.,* 2020).

**EBI** is one of the largest bioinformatics resource providers in Europe ( Brooksbank *et al.*, 2014). The EBI RDF platform includes pairwise orthologous genes information from Ensembl database ( Zerbino *et al.*, 2018). The SPARQL endpoint to access the EBI data is available at https://www.ebi.ac.uk/rdf/services/sparql. For further details, see https://www.ebi.ac.uk/rdf/documentation/ensembl/.

We group the aforementioned databases based on the orthology information type they provide as follows:

I. **Hierarchical Orthologous Groups (HOGs).** The three data sources that provide evolutionary relationship data in RDF to represent HOGs are **OrthoDB**, **MBGD** and **OMA**. Although the RDF data models of MBGD and OMA both rely on the ORTH ontology ( Fernández-Breis *et al.*, 2016), they use different ORTH versions. However, SPARQL queries running over either of the two sources can be formulated in a very similar manner. In the case of OrthoDB, data are organised according to their own internal data model, while also providing cross-references to the UniProt RDF store.
II. **Homologous groups** are sets of homologous genes without any hierarchical grouping (“flat”). All members are homologous to all other members, with no distinction of paralogy or orthology. However, the kind of homologous groups considered in this tutorial do not contain “out-paralogs”—i.e. paralogs which result from gene duplications which took place prior to the last common ancestor of all species in the databases. Furthermore, each homologous group can still be associated with a taxonomic level, which indicates to which species clade its members belong. Example of orthology databases from which we can extract these homologous groups are **OMA**, **OrthoDB** and **MBGD**.
III. **Pairwise orthology**. Apart from the aforementioned orthologous groups, evolutionary data can also be provided in the form of pairwise orthologous genes. Among the sources that provide this type of information in RDF, we consider in this article **EBI**, **OMA**, **OrthoDB** and **MBGD**.

We mention that the SPARQL endpoints of the databases may sometimes be temporarily unavailable, for example, for maintenance purposes. In these cases, the queries provided in this article may not be able to run or may become unresponsive due to the unavailability of the corresponding SPARQL endpoint. Should these issues persist for a longer period of time, it is advised to contact the respective database support through the email address indicated on the SPARQL endpoint webpage.

## Applicable Ontologies

The main existing ontology to represent and structure the orthology information is the Orthology (ORTH) Ontology ( Fernández-Breis, *et al.,* 2016) that is recommended by the Quest for Orthologs Consortium (QfO). The second version of the ORTH ontology is further described at https://github.com/qfo/OrthologyOntology ( de Farias *et al.,* 2017). Both OMA and MBGD rely on the Orthology Ontology. More precisely, OMA uses the ORTH version 2, while MBGD version 1. In contrast, OrthoDB relies on an internal data model while the fragment of EBI relevant to this article mainly reuses the “is orthologous to” pairwise property from the Semanticscience Integrated Ontology (SIO). In the context of the four databases depicted in this article, a way to possibly identify relevant URIs (Uniform Resource Identifiers) for properties of interest such as “in taxon”, “is orthologous to”, but also for taxonomic identifiers etc, is the Ontology Lookup Service (OLS), available online at https://www.ebi.ac.uk/ols/index.

We note below the other main underlying ontologies, controlled vocabularies and taxonomies used by at least one of the four data sources. Further details are depicted in Table 1 in the *Extended data* available in ( Sima & Mendes de Farias, 2020).

- The Gene Ontology (GO) to specify molecular functions, for example.
- The Relation Ontology (part of the Open Biological and Biomedical Ontology (OBO) Foundry) for properties such as “in taxon”.
- SIO for properties such as “protein encoded by” and “gene encodes protein”.
- ENSEMBL for gene identifiers.
- The National Center for Biotechnology Information (NCBI) taxonomy.
- Dublin Core Initiative Metadata (DCMI) terms to state identifiers and descriptions in OMA and MBGD databases.
- UniProt core ontology – for scientific/common names of species, as well as cross-references.

In the Section “Data Models” we provide a more detailed introduction to how each of the four data sources considered in this article structures orthology data. We illustrate through concrete examples the commonalities as well as differences between the data sources.

### Data models

In this section we provide a brief introduction to the data models of the orthology databases considered in this article, in order to facilitate the understanding of the SPARQL queries presented in the Protocol Section.

We first present a simple example to illustrate how SPARQL queries can be formulated, starting from a given data model. 

**Figure 1.**
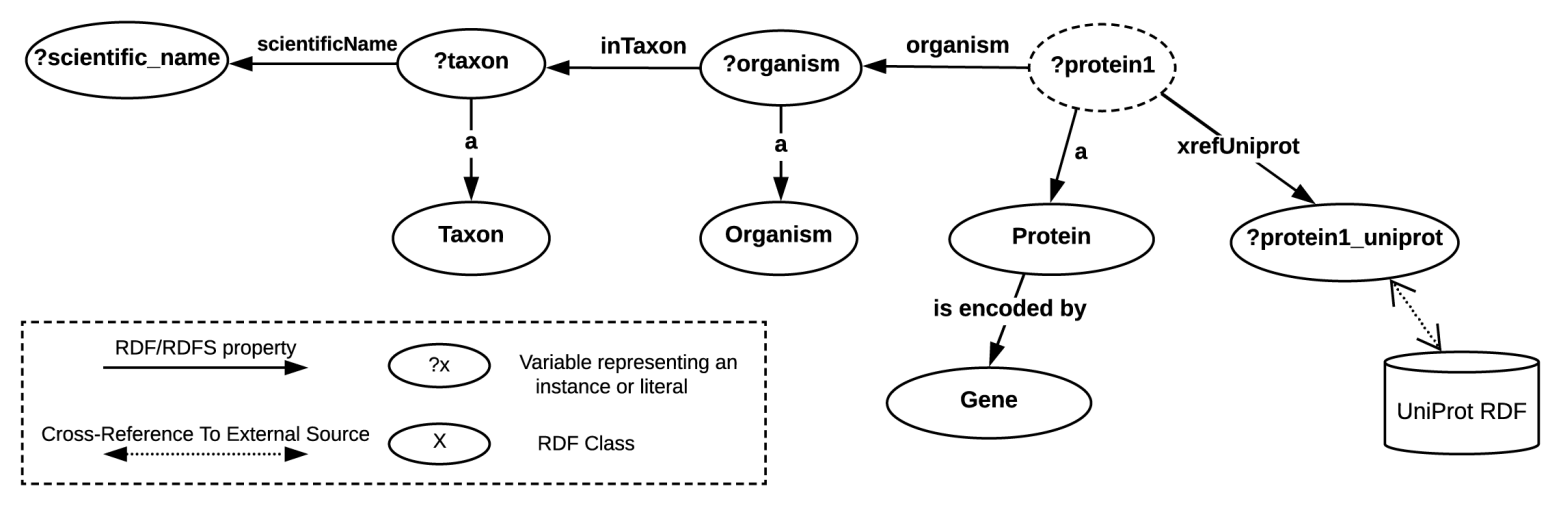
Simplified query graph that can be used as support for writing SPARQL queries to extract relevant information, such as proteins in a particular species.

Figure 1 illustrates a simplified graph abstraction of an RDF data model targeting proteins and genes that encode these proteins, and their related species (taxa). Furthermore, the model includes cross-references to the corresponding UniProt entries. These cross-references can be useful in formulating federated SPARQL queries. Federated queries can retrieve information from multiple RDF data sources, using the UniProt entry as an intersection ("join point"). 

Figure 1 can be used as a guide to formulate simple SPARQL queries that aims to answer questions of interest. Such questions can be related to, for example, proteins found in a given species, or those corresponding to a specific UniProt accession number. For readability, we omitted specific namespaces and exact URIs. Instead, we use the human readable labels of properties, such as "in taxon". The URIs that represents these terms (e.g. Protein, Gene, in taxon) depend on the underlying database being queried. Nevertheless, if the database is reusing existing vocabularies to model their data, a reference for mapping these terms to their corresponding identifiers (URIs), is the Ontology Lookup Service (OLS), and the Linked Open Vocabularies (LOV) https://lov.linkeddata.es. For example, searching for "in taxon" in OLS will result in the first answer returned being the OBO URI http://purl.obolibrary.org/obo/RO_0002162.

An example question for which the corresponding SPARQL query can be formulated, based on the simplified query graph in Figure 1, could be:

      “What are Rattus Norvegicus proteins available in the database?”

In order to retrieve this information, we can start from the ?protein1 variable, of type Protein, illustrated in the top right corner of the figure, which we need to connect to the taxon scientific name ("Rattus Norvegicus") via “organism”. Note that the SPARQL users can define variable names as they want by following the SPARQL syntax where the question mark (?) means that is a variable. Following the arrows from ?protein1 to ?taxon (in Figure 1, from right to left) we can formulate the following SPARQL query, which can be executed in the OMA SPARQL endpoint:

PREFIX up: <http://purl.uniprot.org/core/>

PREFIX orth: <http://purl.org/net/orth#>

       SELECT ?protein1 WHERE {

              ?protein1 a orth:Protein.

              ?protein1 orth:organism ?organism.

              ?organism <http://purl.obolibrary.org/obo/RO_0002162> ?taxon.

              ?taxon up:scientificName "Rattus norvegicus".}

Changing the target species of interest only requires changing the corresponding text in between quotes. For example, to find the human proteins available in the data source, change "Rattus norvegicus" to "Homo sapiens" (note: the query is case-sensitive).

Any additional information can be retrieved by adding the corresponding triple pattern to the query, while also making sure to add any relevant variables to the select statement. For example, to also retrieve the corresponding UniProt entries for the proteins, we can apply the following modification (changes highlighted in bold, see last line of the query statement):

PREFIX up: <http://purl.uniprot.org/core/>

PREFIX orth: <http://purl.org/net/orth#>

PREFIX lscr: <http://purl.org/lscr#>

       SELECT ?protein1 ?uniprot_entry WHERE {

              ?protein1 a orth:Protein.

              ?protein1 orth:organism ?organism.

              ?organism <http://purl.obolibrary.org/obo/RO_0002162> ?taxon.

              ?taxon up:scientificName "Rattus norvegicus".

**              ?protein1 lscr:xrefUniprot ?uniprot_entry.**}

For a more complete introduction to SPARQL and RDF in the context of biological databases see ( Sima *et al.,* 2019). Next we will introduce the data models of the orthology databases considered in this article.

**Figure 2.**
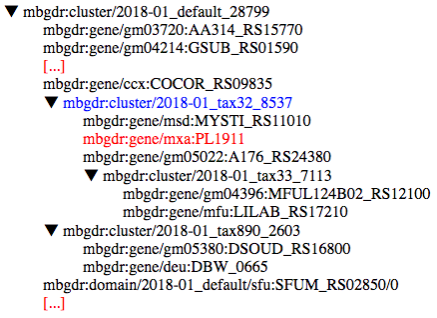
A fragment of the hierarchical orthologous cluster no. 28799 in MBGD. A cluster can consist of genes, domains (sub-genes) or further nested orthologous clusters. Multiple levels of the hierarchy may need to be traversed recursively in order to reach a given orthologous gene. For example, the gene mxa:PL1911 (highlighted in red) can be reached through the member orthologous cluster 2018-01_tax32_8537 (shown in blue). This can be achieved in SPARQL through a recursive graph pattern, using the hasHomologous* property path ^1^ - a graphical abstraction of the RDF representation is provided in Figure 3.

Figure 2 illustrates a few of the members of a HOG, the main data structure in MBGD. In particular, this MBGD cluster has the identifier *28799*. Members of an MBGD orthologous cluster can be either genes, domains or other clusters. These nested orthologous clusters are built at specific taxonomic levels in the hierarchy. For example, the cluster highlighted in blue in Figure 2 was built at taxonomic level 32, *Myxococcus*. The hierarchy needs to be traversed in order to reach genes, such as *mxa:PL1911* that is highlighted in red in Figure 2, or domains (sub-gene level) which belong to an orthologous cluster at a given taxonomic level.

The RDF model is more suitable for representing such hierarchical data structures than the relational data model ( Sima *et al.*, 2019) given that RDF is a graph data model. Moreover, querying orthology RDF data can benefit from SPARQL 1.1 *recursive* graph patterns such as property paths. The main construct in SPARQL required to retrieve the orthologous genes of a gene of interest X will then be the following recursive pattern:

*?hog_cluster a orth:OrthologsCluster.*

?hog_cluster orth:hasHomologous* ?gene_X.

?hog_cluster orth:hasHomologous* ?orthologous_gene_Y.

For example, we can replace *?gene_X* with the URI of the human Hemoglobin Subunit Beta (HBB) gene, namely: < http://mbgd.genome.ad.jp/rdf/resource/gene/hsa:HSA_4504349>, which would enable retrieving all orthologs of human HBB through the *?orthologous_gene_Y* variable. The asterisk (*) following the “ *orth:hasHomologous*” property indicates that this property should be matched *recursively*.

**Figure 3.**
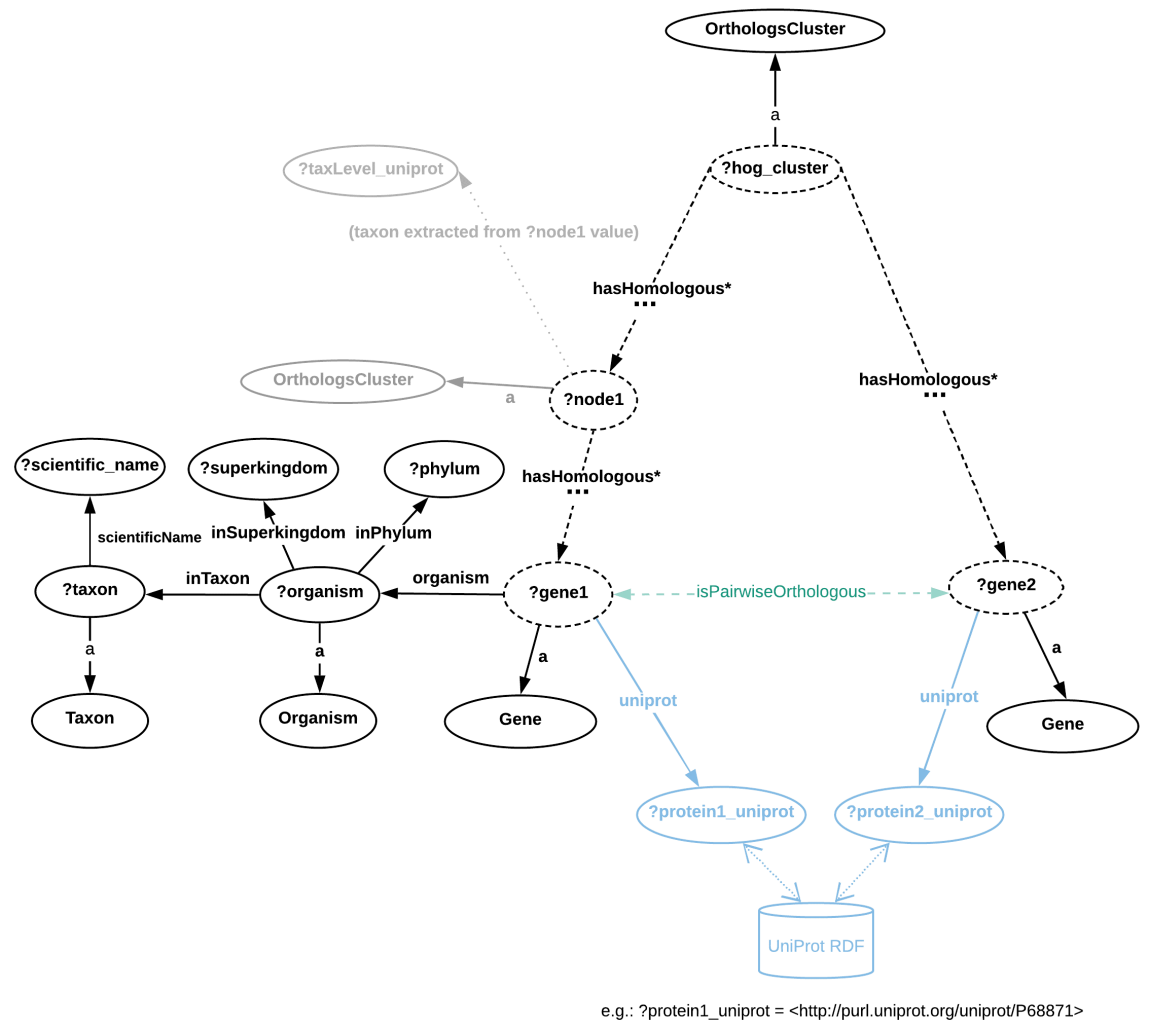
Directed graph abstraction of a portion of the MBGD RDF graph related to hierarchical orthologous groups. In Figure 3, nodes are either classes or variables, and edges are RDF properties. The terms preceded by a question mark (e.g. *?gene1*) represent variables assigned with either zero or more literals or URIs. Dashed edges illustrate the orth:hasHomologous property that can be stated zero or more times, recursively. URI prefixes were omitted. MBGD is gene-centric and contains taxonomic ranges where HOGs are built are not directly available in RDF - in some cases these can be extracted from the cluster URI (e.g. http://mbgd.genome.ad.jp/rdf/resource/cluster/2018-01_tax32_8537 corresponds to taxonomic identifier 32, Myxococcus). By contrast, the taxonomic information per gene entry is richer in MBGD than in OMA, including explicit Superkingdom and Phylum information. Example SPARQL queries based on this graph abstraction are provided in the “Protocols” section, as well as in the accompanying Jupyter notebook. The pairwise orthology information is not directly available (e.g. through an RDF property), but can be extracted from the Orthologs Cluster (to highlight this, the “isPairwiseOrthologous” is shown in green with a dashed arrow).

Based on Figure 3, SPARQL queries can be formulated by following the directions and labels of arrows in order to formulate triple patterns. For example, to retrieve all genes (i.e. instances of the *orth:Gene* class) of a given HOG, we can follow the graph structure from root to leaf members by performing the query as shown in the code fragment below. In other words, the *?gene1* variable values illustrated as the left-side member in the cluster *?hog_cluster* (see Figure 3).

This SPARQL query will retrieve all the genes in the MBGD Hierarchical Orthologous Group represented with the identifier 28799.

PREFIX orth: <http://purl.org/net/orth#>

PREFIX cluster-id:<http://mbgd.genome.ad.jp/rdf/resource/cluster/>

SELECT ?hog_cluster ?gene1 WHERE {

  VALUES ?hog_cluster {cluster-id:2018-01_default_ **28799**}

  ?hog_cluster a orth:OrthologsCluster.

  ?hog_cluster orth:hasHomologous* ?gene1.

  ?gene1 a orth:Gene. }

**Figure 4.**
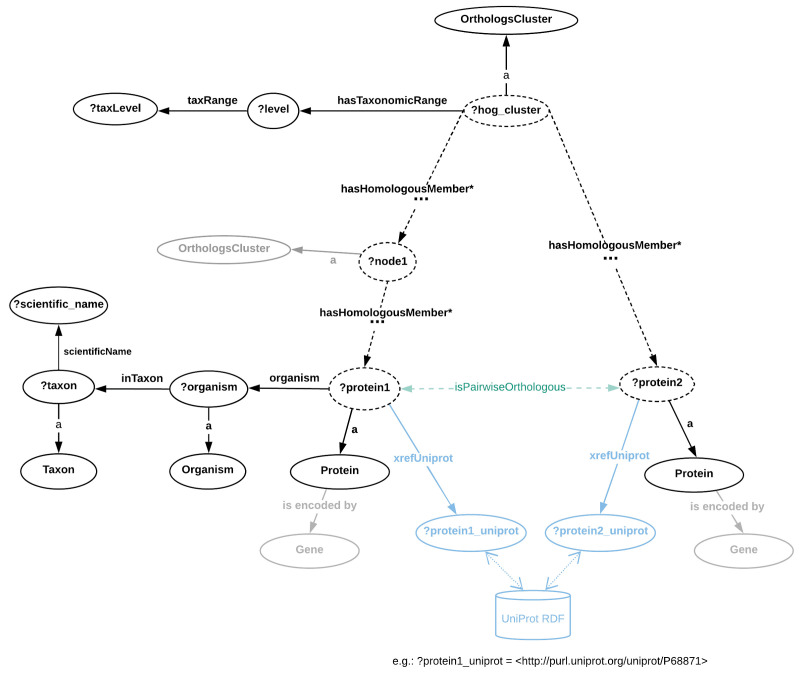
Directed graph abstraction of a portion of the OMA RDF graph related to hierarchical orthologous groups. In Figure 4, dashed edges illustrate the orth:hasHomologousMember property that can be stated zero or more times, recursively. OMA is protein-centric, however the corresponding genes that encode the proteins are also available in RDF through the "is encoded by" property (a cross-reference to Ensembl identifiers is also provided). Furthermore, the taxonomic ranges where HOGs were built are asserted through the “hasTaxonomicRange” property. The pairwise orthology information is not directly available (e.g. through an RDF property), but can be extracted from the Orthologs Cluster (to highlight this, the “isPairwiseOrthologous” is shown in green with a dashed arrow). Note: URI prefixes were omitted.

Similarly, the HOG structure in OMA is abstracted in Figure 4. Both figures can be used as a guide in formulating SPARQL queries, by following the directions of the arrows in order to formulate triple patterns. Since both the MBGD and the OMA models rely on the ORTH ontology ( Fernández-Breis *et al.*, 2016), the two graph structures are very similar and therefore SPARQL queries can be formulated with only minor differences for both data sources. 

**Figure 5.**
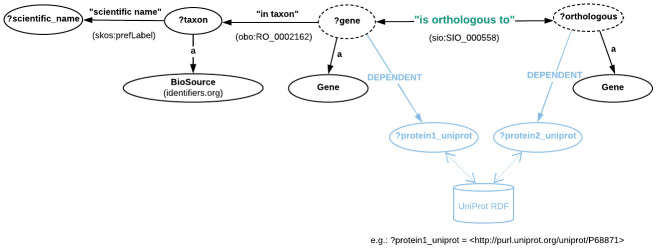
Directed graph abstraction of a portion of the EBI RDF graph related to pairwise orthologous genes. Moreover, as opposed to the RDF representations in OMA and MBGD, here the pairwise orthology is explicitly asserted through the “is orthologous to” property (more precisely, http://semanticscience.org/resource/SIO_000558) as shown in Figure 5. However, there is no information available regarding orthologous clusters. Moreover, the Gene class here is in fact the OBO (not ORTH) class, i.e. http://purl.obolibrary.org/obo/SO_0000704. Instances of these genes can be specified either through their cross-reference to UniProt (the http://rdf.ebi.ac.uk/terms/ensembl/DEPENDENT property) or directly through their ENSEMBL identifier, by fixing the value of ?gene to the concatenation of http://rdf.ebi.ac.uk/resource/ensembl/ and the corresponding Ensembl identifier. Finally, the taxonomic identifiers are provided via instances of the BioSource class, http://www.biopax.org/release/biopax-level3.owl#BioSource.

Figure 5 illustrates the data model of the portion of the EBI RDF graph describing orthology information. In contrast to OMA, OrthoDB and MBGD, EBI only provides pairwise orthologous genes.

**Figure 6.**
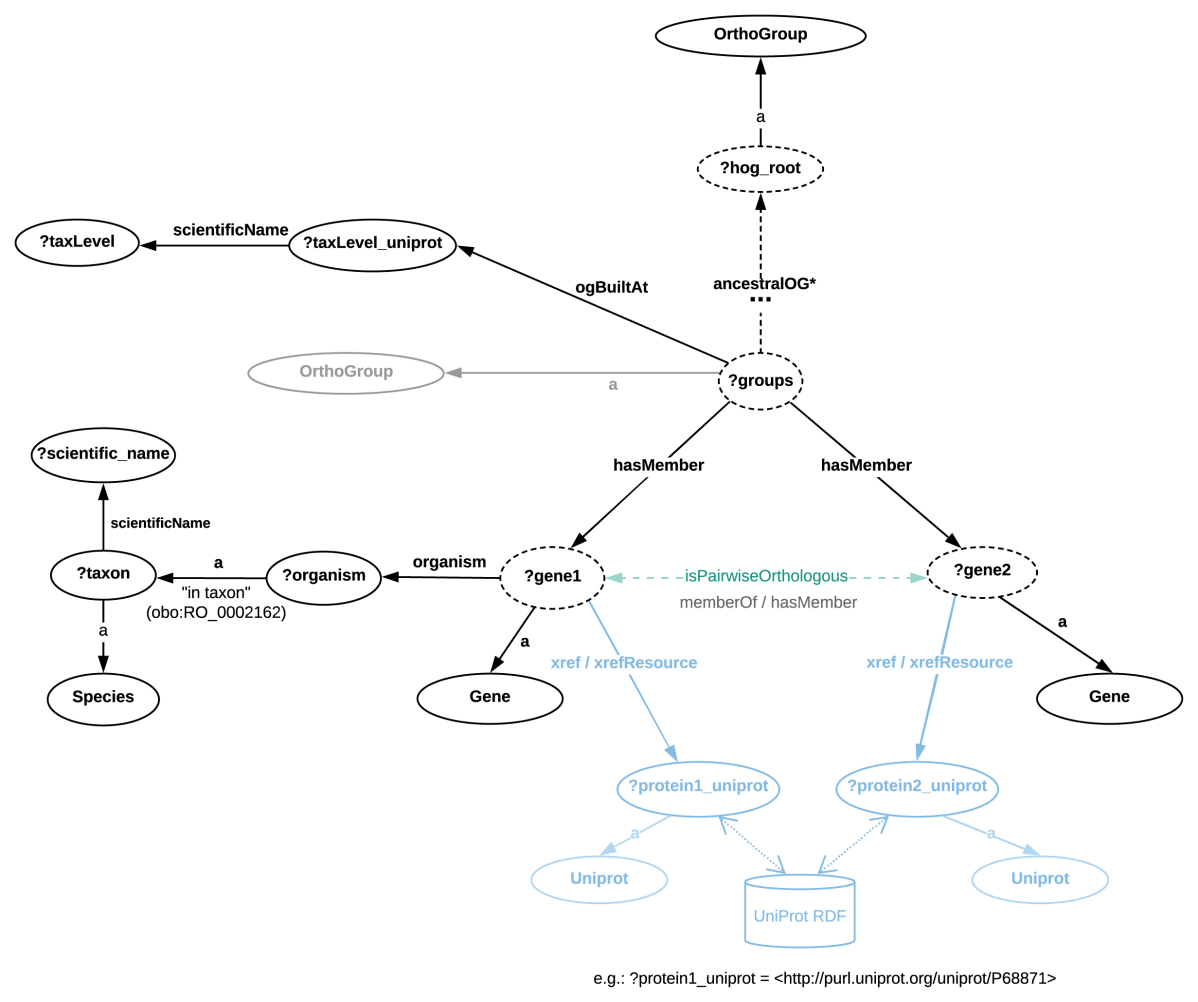
Directed graph abstraction of a portion of the OrthoDB RDF graph related to orthologous groups. Note that the abstract relation *“?gene1 isPairwiseOrthologous ?gene2”* is derived by considering the concrete property path *“?gene1 :memberOf / :hasMember ?gene2”* that further implies the following joint triples: *“?gene1 :memberOf ?group. ?group :hasMember ?gene2.”*. In Figure 6, genes are direct members of OrthoGroups built at a given taxonomic level (Clade), e.g. Cyanobacteria, available through the "ogBuiltAt" property. The cross-references to UniProt (as well as Ensembl and Entrez) are available through a 2-triple pattern (for examples see “Protocols” section).

Figure 6 illustrates the structure of Orthologous Groups in the OrthoDB RDF. Here, genes are direct members of OrthoGroups built at a given taxonomic level (Clade), e.g. *Cyanobacteria*. We mention that OrthoDB provides information in RDF, including sequence length, number of exons for gene members, as well as evolutionary rates, functional category and others for orthologous groups (for more details see *Extended data* in ( Sima & Mendes de Farias, 2020)).

### Choosing the relevant target gene identifiers in RDF (URIs)

One of the challenges in formulating SPARQL queries is identifying the relevant URIs for the resources of interest. In the case of queries targeting the orthology domain, the resources of interest will usually consist of target genes, for which the orthologous genes need to be retrieved from the RDF data store. A simple way to obtain the relevant URIs in the case of the four data sources considered in this article, is to start from the UniProt accession numbers for the target gene of interest. This accession number can be identified through the online search interface of UniProt at www.uniprot.org, by searching for the gene name of interest, for example, “HBB” (or “human HBB”). The column “Entry” in the result page contains the corresponding UniProt accession number. From here, the URI can be obtained by concatenating the UniProt namespace prefix: http://purl.uniprot.org/uniprot/ with this accession number. 

For example, in the case of human HBB, the URI will be http://purl.uniprot.org/uniprot/P68871. Using this information and the cross-reference properties available in each of the four databases (OMA, OrthoDB, MBGD and OrthoDB), the appropriate SPARQL queries can be formulated, according to the examples shown previously in Figure 3– Figure 6. In the “Protocols” section we give concrete examples of queries for each of the four data sources, which can be directly executed in the corresponding SPARQL endpoints.

## Protocols – SPARQL queries

In this section, we provide four protocols to (i) retrieve pairwise orthologs through SPARQL queries from EBI, OMA, MBGD, as well as (ii) homologous groups from OMA, MBGD and OrthoDB (iii) restrict the search to a given taxonomic level (iv) perform meta-analyses across multiple data sources providing orthology information, and aggregations using the entire data available in a given data source. All protocols presented below are included in the accompanying Jupyter notebook.

For the sake of simplicity, genes are identified with either their Ensembl identifiers or their cross-reference to the UniProt accession number. In this article, we assume the reader already knows the UniProt primary accession number of the searched gene. In general, this number can be found by searching for the corresponding gene name in the UniProt webpage, for example, “HBB” (i.e. “hemoglobin subunit beta”). As a reminder, the UniProt protein identifier in RDF is a URI composed of the UniProt accession number (e.g. P68871) appended to the UniProt namespace prefix: http://purl.uniprot.org/uniprot/. For instance, in the case of the human HBB gene, the corresponding URI identifier is http://purl.uniprot.org/uniprot/P68871.

### Protocol 1: Retrieve pairwise orthologs (OrthoDB, EBI, OMA, MBGD)

In this protocol we illustrate the basic task of retrieving the pairwise orthologs of a given gene, for example the HBB (Hemoglobin subunit beta) human gene. This is illustrated on the four orthology databases that provide pairwise orthology information in RDF: OrthoDB, EBI, OMA and MBGD. The corresponding SPARQL queries to retrieve the pairwise orthologs can be formulated as shown below. We note that the resulting orthologs are also provided using their “clickable” cross-reference link to UniProt. This can directly be used to find out more information about the resulting genes (e.g. name, location, expression) and has the added advantage that results originating from different orthology databases can then be compared against each other. All Protocol 1 queries retrieve a set of 2-tuples *(?a, ?b)*, where *?a* is the gene given as an input to look for its orthologous genes, which are assigned to *?b*. Therefore, the 2-tuple *(?a, ?b)* set represents the binary relation *“?a is orthologous to ?b*”. In all queries except for Q2, these genes are represented with UniProt entries, more specifically, UniProt URIs (see subsection “Choosing the relevant target gene identifiers in RDF” for further details). In Q2, the input gene is represented with an Ensembl gene URI as depicted in b).

a) Retrieving OrthoDB pairwise orthologs

The following code fragment shows the SPARQL query to retrieve pairwise orthologs of the human HBB gene from the OrthoDB database. The following link http://purl.org/orthology/q0 is provided to directly execute the query at the OrthoDB SPARQL endpoint. We denote this query as Q0.

PREFIX : <http://purl.orthodb.org/>

SELECT DISTINCT ?protein1 ?protein2{

VALUES(?protein1){(<http://purl.uniprot.org/uniprot/P68871>)}

              ?og a :OrthoGroup.

              ?gene1 :memberOf ?og.

              ?og :hasMember ?gene2.

              ?gene1 :xref ?xref1.

              ?xref1 :xrefResource ?protein1.

              ?gene2 :xref ?xref2.

              ?xref2 :xrefResource ?protein2.

              ?protein2 a :Uniprot. }

The HBB gene is also represented in the latest code fragment with the UniProt URI http://purl.uniprot.org/uniprot/P68871. However, differently to other databases, OrthoDB states cross-references to UniProt by joining three triple patterns: ?g :xref ?x and ?x :xrefResource ?p and ?p a :Uniprot.

*b) Retrieving EBI pairwise orthologs*

The following code fragment, which we will denote as Q1, depicts a SPARQL query to retrieve pairwise orthologs of the human HBB gene from Ensembl dataset at the EBI RDF platform (see Figure 5 for the respective data schema).

You can execute this query directly at the EBI SPARQL endpoint by clicking on the following link: http://purl.org/orthology/q1.

PREFIX obo: <http://purl.obolibrary.org/obo/>

PREFIX sio: <http://semanticscience.org/resource/>

PREFIX ensembl: <http://rdf.ebi.ac.uk/resource/ensembl/>

PREFIX ensemblterms: <http://rdf.ebi.ac.uk/terms/ensembl/>

SELECT DISTINCT ?gene_uniprot_uri ?ortholog_uniprot_uri {

  VALUES(?gene_uniprot_uri){(<http://purl.uniprot.org/uniprot/ **P68871**>)}

  ?gene sio:SIO_000558 ?ortholog . #«is orthologous to»

  ?gene obo:RO_0002162 ?taxon . #«in taxon»

  ?ortholog obo:RO_0002162 ?ortholog_taxon.

  ?ortholog ensemblterms:DEPENDENT ?ortholog_uniprot_uri.

  ?gene ensemblterms:DEPENDENT ?gene_uniprot_uri.

  FILTER(?taxon != ?ortholog_taxon

               &&

STRSTARTS(STR(?ortholog_uniprot_uri),"http://purl.uniprot.org/uniprot/") )}

The HBB gene is represented with the UniProt URI http://purl.uniprot.org/uniprot/P68871. To retrieve the orthologs of other genes, we can replace this URI with one that corresponds to another gene, such as human INS (i.e. http://purl.uniprot.org/uniprot/P01308). We can also provide a set of URIs enclosed with parentheses such as follows: VALUES(?gene_uniprot_uri) {(<http://purl.uniprot.org/uniprot/P68871>)(<http://purl.uniprot.org/uniprot/P01308>) }. The sio:SIO_000558 is the « is orthologous to » property, while the obo:RO_0002162 represents the « in taxon » property (see Figure 5).

We note here that not all EBI gene entries have an assigned cross-reference to UniProt. For example, “ *ENSG00000139618*” identifies an Ensembl gene for which the UniProt cross-reference is missing from the EBI RDF platform. In this case, the previous SPARQL query can be adapted, by assigning in the VALUES statement of the query, the ?gene variable to the corresponding Ensembl identifier, as depicted in the following code fragment, which we denote as Q2. Q2 can be executed at the EBI SPARQL endpoint by clicking on the following link: http://purl.org/orthology/q2.

PREFIX obo: <http://purl.obolibrary.org/obo/>

PREFIX sio: <http://semanticscience.org/resource/>

PREFIX ensembl: <http://rdf.ebi.ac.uk/resource/ensembl/>

PREFIX ensemblterms: <http://rdf.ebi.ac.uk/terms/ensembl/>

SELECT DISTINCT ?gene ?ortholog_uniprot_uri {

   VALUES(?gene){(ensembl: **ENSG00000139618**)}

   ?gene sio:SIO_000558 ?ortholog.

   ?gene obo:RO_0002162 ?taxon.

   ?ortholog obo:RO_0002162 ?ortholog_taxon.

   ?ortholog ensemblterms:DEPENDENT ?ortholog_uniprot_uri.

   ?gene ensemblterms:DEPENDENT ?gene_uniprot_uri.

FILTER(?taxon != ?ortholog_taxon &&

STRSTARTS(STR(?ortholog_uniprot_uri),"http://purl.uniprot.org/uniprot/") )}

This code fragment illustrates the SPARQL query to retrieve orthologs for the human BRCA2 gene from the Ensembl dataset. The BRCA2 gene is represented with the UniProt URI ensembl:ENSG00000139618 where ensembl is a prefix that replaces http://rdf.ebi.ac.uk/resource/ensembl/. To retrieve the orthologs of other genes, we can replace ensembl:ENSG00000139618 with a URI that corresponds to another gene such as human INS (i.e. ensembl:ENSG00000254647). We can also provide a set of URIs enclosed with parentheses such as follows: VALUES(?gene){(ensembl:ENSG00000139618)(ensembl:ENSG00000254647)}.

*c) Retrieving OMA pairwise orthologs*

The following code fragment shows a SPARQL query to retrieve pairwise orthologs of the human HBB gene which are derived from the HOGs in the OMA database (see Figure 4 for the respective data schema). The following link http://purl.org/orthology/q3 is provided to directly execute the query at the OMA SPARQL endpoint webpage. We denote this query as Q3.

PREFIX oma: <http://omabrowser.org/ontology/oma#>

PREFIX orth: <http://purl.org/net/orth#>

PREFIX sio: <http://semanticscience.org/resource/>

PREFIX lscr: <http://purl.org/lscr#>

SELECT DISTINCT ?protein1 ?protein2 {

VALUES(?protein1){(<http://purl.uniprot.org/uniprot/ **P68871**>)}

              ?cluster a orth:OrthologsCluster.

              ?cluster orth:hasHomologousMember ?node1.

              ?cluster orth:hasHomologousMember ?node2.

              ?node1 orth:hasHomologousMember* ?protein_OMA_1.

              ?node2 orth:hasHomologousMember* ?protein_OMA_2.

              ?protein_OMA_1 lscr:xrefUniprot ?protein1.

              ?protein_OMA_2 lscr:xrefUniprot ?protein2.

FILTER(?node1 != ?node2)}

The HBB gene is represented in this code fragment with the UniProt URI http://purl.uniprot.org/uniprot/P68871. More precisely, in OMA the lscr:xrefUniprot represents the Cross-reference to UniProt.

*d) Retrieving MBGD pairwise orthologs*

In a similar manner to the previous code fragment, the following depicts a SPARQL query to retrieve pairwise orthologs of the human HBB gene which are derived from the HOGs in the MBGD database (see Figure 3 for the respective data schema). To obtain the results for this query, denoted as Q4, by using the MBGD SPARQL endpoint, we can click on the following link: http://purl.org/orthology/q4.

PREFIX mbgdr: <http://mbgd.genome.ad.jp/rdf/resource/>

PREFIX mbgd: <http://purl.jp/bio/11/mbgd#>

PREFIX orth: <http://purl.org/net/orth#>

SELECT ?protein1 ?protein2

WHERE {

VALUES(?protein1){ (<http://purl.uniprot.org/uniprot/P68871>)}

              ?cluster a orth:OrthologsCluster.

              ?cluster orth:hasHomologous ?node1.

              ?cluster orth:hasHomologous ?node2.

              ?node1 orth:hasHomologous* ?gene1.

              ?node2 orth:hasHomologous* ?gene2.

              ?gene1 mbgd:uniprot ?protein1.

              ?gene2 mbgd:uniprot ?protein2.

FILTER(?node1 != ?node2)}

The HBB gene is represented again with the UniProt URI http://purl.uniprot.org/uniprot/P68871. In the case of MBGD, the mbgd:uniprot represents the cross-reference to UniProt.

### Protocol 2: Retrieve homologous groups

In this protocol we illustrate the task of retrieving the non-hierarchical homologous groups of a target gene, such as the human HBB gene. In addition, we restrict the search to a specific taxonomic level, for example, “only at the primates level”. In other words, we depict how to retrieve the homologous groups at a given taxonomic level and including a given gene represented as a UniProt URI. Note that the same query can be executed only providing one of the inputs (i.e. either the taxonomic level or gene). However, it can take longer to return all results or may not even be executed due to runtime constraints at the original databases. The members of a homologous group can be either paralogous or orthologous to one another.

*a) Retrieving OMA Homologous Groups derived from the HOGs*

The following code fragment denoted as Q5 illustrates the SPARQL query to retrieve homologous groups (i.e. clusters) that contains the human HBB gene in the OMA database. To execute it directly at the OMA SPARQL endpoint webpage at https://sparql.omabrowser.org/lode/sparql, you can click on the following link: http://purl.org/orthology/q5.

PREFIX lscr: <http://purl.org/lscr#>

PREFIX orth: <http://purl.org/net/orth#>

SELECT DISTINCT ?cluster ?protein2_OMA_URI ?protein2_uniprot_URI ?tax_name {

  VALUES(?protein1_uniprot_URI){(<http://purl.uniprot.org/uniprot/P68871>)}

  VALUES(?tax_name){("Primates")}

  ?cluster a orth:OrthologsCluster.

  ?cluster orth:hasHomologousMember* ?protein_OMA_1.

  ?cluster orth:hasHomologousMember* ?protein2_OMA_URI.

  ?protein_OMA_1 a orth:Protein.

  ?protein2_OMA_URI a orth:Protein.

  ?protein_OMA_1 lscr:xrefUniprot ?protein1_uniprot_URI.

  OPTIONAL{?protein2_OMA_URI lscr:xrefUniprot ?protein2_uniprot_URI.}

  ?cluster orth:hasTaxonomicRange ?tax.

  ?tax orth:taxRange ?tax_name. }

In this code fragment, the HBB gene is represented with its related UniProt entry (i.e. the UniProt URI http://purl.uniprot.org/uniprot/P68871). To retrieve the clusters that have other genes, we can replace this URI with one that corresponds to another gene such as human INS (i.e. http://purl.uniprot.org/uniprot/P01308). We can also provide a set of URIs enclosed with parentheses such as follows:

VALUES(?protein1_uniprot_URI) {(<http://purl.uniprot.org/uniprot/P68871>) (<http://purl.uniprot.org/uniprot/P01308>)}. Similarly, we can change the taxonomic level of reference as follows: VALUES(?tax_name) {("Hominoidea")}.

In further details, the Q5 query retrieves a set of 4-tuples (?cluster, ?protein2_OMA_URI, ?protein2_uniprot_URI, ?tax_name). The ?cluster variable represents the homologous group built at a taxonomic level of reference (i.e. ?tax_name) that contains the gene represented with a given UniProt entry (e.g. P68871). The ?protein2_OMA_URI and ?protein2_uniprot_URI variables are assigned the homologous genes defined as OMA and UniProt entries, respectively. These genes belong to the same homologous group (i.e. ?cluster). Because of the fact that the SPARQL results are in a tabular form, to solely retrieve the members of a homologous group that are represented as a UniProt entry, we mainly need to project the ?protein2_uniprot_URI. To do so, we have to replace the line containing the SELECT keyword in Q5 with the following instruction: SELECT DISTINCT ?protein2_uniprot_URI.

*b) MBGD Homologous Groups derived from the HOGs*

The HOGs in MBGD do not provide explicit taxonomic levels at the root level of a HOG. However, the taxon NCBI identifiers of subHOGs (i.e. sublevels) can be extracted in some cases from the cluster URI. Since this requires more advanced knowledge of SPARQL (in particular, for parsing the cluster URIs), we only make it available as part of the *Extended data* in ( Sima & Mendes de Farias, 2019).

*c) OrthoDB Homologous Groups*

The following code fragment denoted as Q6 retrieves homologous groups that contains the human HBB gene identified with its corresponding UniProt entry accession number P68871. The query can be executed at the OrthoDB SPARQL endpoint webpage at https://sparql.orthodb.org. To inspect the results, we can execute Q6 by accessing the following link: http://purl.org/orthology/q6

PREFIX orthodb: <http://purl.orthodb.org/>

PREFIX up: <http://purl.uniprot.org/core/>

SELECT DISTINCT ?group ?species_name ?protein1_uniprot ?gene1 ?taxLevel_uniprot ?taxLevel WHERE {

              VALUES ?protein2_uniprot {<http://purl.uniprot.org/uniprot/P68871>}

              VALUES ?taxLevel {"Primates"}

              ?gene2 a orthodb:Gene.

              ?gene2 orthodb:memberOf ?group.

              ?gene1 a orthodb:Gene.

              ?gene1 orthodb:memberOf ?group.

              ?gene1 up:organism ?organism.

              ?organism a ?taxon.

              ?taxon up:scientificName ?species_name.

              ?group orthodb:ogBuiltAt ?taxLevel_uniprot.

              ?taxLevel_uniprot up:scientificName ?taxLevel.

              ?gene2 orthodb:xref ?xref2.

              ?xref2 orthodb:xrefResource ?protein2_uniprot.

              ?protein2_uniprot a orthodb:Uniprot.

              ?gene1 orthodb:xref ?xref.

              ?xref a orthodb:Xref.

              OPTIONAL{

              ?xref orthodb:xrefResource ?protein1_uniprot.

              ?protein1_uniprot a orthodb:Uniprot.}

} ORDER BY ?group, ?taxLevel

This SPARQL query will retrieve flat homologous groups that contains the human HBB gene in OrthoDB. More specifically, these homologous groups are indeed orthologous groups, similarly to groups in which all genes are related to each other by pairwise orthologous relations ( Zahn-Zabal *et al.,* 2020). Moreover, the HBB gene is represented with its related UniProt entry (i.e. the UniProt URI http://purl.uniprot.org/uniprot/P68871).

In more details, the Q6 query retrieves a set of 6-tuples (?group ?species_name ?protein1_uniprot ?gene1 ?taxLevel_uniprot ?taxLevel ). The ?group variable represents the homologous group built at a taxonomic level of reference (i.e. ?taxLevel) that contains the gene represented with a given UniProt entry (e.g. P68871). The ?gene1 and ?protein1_uniprot variables are assigned the orthologous genes defined as OrthoDB and UniProt entries, respectively. These genes belong to the same homologous group (i.e. ?group). In addition, ?species_name and ?taxLevel_uniprot variables are assigned, respectively, a species scientific name where ?gene1 is found, and ?taxLevel_uniprot is the corresponding UniProt URI of a ?taxLevel value (i.e. a taxonomic level name, e.g. “Primates”). To solely retrieve the members of an OrthoDB orthologous group that are represented as UniProt entries, we just need to project the ?protein1_uniprot variable in the SELECT query form.

### Protocol 3: Retrieve Hierarchical Orthologous Groups (HOG)

In this protocol we show how to retrieve the HOGs containing a target gene, such as the human HBB gene, in the three orthology databases OMA, MBGD and OrthoDB. The Ensembl dataset in the EBI RDF platform is not considered because it does not provide HOG information. 

*a) Retrieving HOGs from OMA*

The following code fragment denoted as Q7 retrieves hierarchical orthologous groups (HOGs) that contains a gene identified with the UniProt entry accession number P68871. Q7 can be executed at the OMA SPARQL endpoint webpage at https://sparql.omabrowser.org/lode/sparql. The query along with its results are available at http://purl.org/orthology/q7.

PREFIX obo: <http://purl.obolibrary.org/obo/>

PREFIX orth: <http://purl.org/net/orth#>

PREFIX taxon: <http://purl.uniprot.org/taxonomy/>

PREFIX up: <http://purl.uniprot.org/core/>

PREFIX lscr: <http://purl.org/lscr#>

SELECT DISTINCT ?root_hog ?species_name ?protein1_uniprot (?protein1 as

                                       ?protein1_OMA) ?taxLevel {

              VALUES ?protein2_uniprot {<http://purl.uniprot.org/uniprot/P68871>}

              ?root_hog obo:CDAO_0000148 ?hog_cluster. #has_Root

              ?hog_cluster orth:hasHomologousMember* ?node1.

              ?node1 a orth:OrthologsCluster.

              ?node1 orth:hasTaxonomicRange ?level.

              ?level orth:taxRange ?taxLevel.

              ?node1 orth:hasHomologousMember* ?protein1.

              ?hog_cluster orth:hasHomologousMember* ?protein2.

              ?protein1 a orth:Protein.

              ?protein1 orth:organism ?organism.

              ?organism obo:RO_0002162 ?taxon.

              ?taxon up:scientificName ?species_name.

              OPTIONAL{?protein1 lscr:xrefUniprot ?protein1_uniprot}.

              ?protein2 a orth:Protein.

              ?protein2 lscr:xrefUniprot ?protein2_uniprot.

} ORDER BY ?taxLevel

This SPARQL query will retrieve the root hierarchical orthologous group that contain the human HBB gene in the OMA database. The HBB gene is represented with its related UniProt entry (i.e. the UniProt URI http://purl.uniprot.org/uniprot/P68871). More specifically, the Q7 query retrieves a set of 5-tuples (?root_hog, ?species_name, ?protein1_uniprot, ?protein1_OMA, ?taxLevel). The ?root_hog variable represents the root HOG (i.e. the deepest common ancestor for all the present species) that contains the gene represented with a given UniProt entry (e.g. P68871). The ?protein1_OMA and ?protein1_uniprot variables are assigned the genes defined as OMA and UniProt entries, respectively. These genes belong to the same root HOG (i.e. ?root_hog). In addition, ?species_name and ?taxLevel variables are assigned, respectively, the species scientific name and the taxonomic level (e.g. “Tetrapoda”) where the gene is found. Moreover, the taxonomic levels implicitly represent speciation events and ancestral genes in the context of HOGs.

*b) Retrieving HOGs from MBGD*

The SPARQL query to retrieve HOGs from MBGD is similar to the previous query over OMA and therefore we make it available as *Extended data* in ( Sima & Mendes de Farias, 2020). As a reminder, although both the OMA and MBGD databases rely on different versions of the ORTH ontology, they structure their HOG data similarly.

*c) Retrieving HOGs from OrthoDB*

The following code fragment denoted as Q8 retrieves hierarchical orthologous groups that contain the human HBB gene in the OrthoDB database. The HBB gene is represented with its related UniProt entry (i.e. the UniProt URI http://purl.uniprot.org/uniprot/P68871). The query can be executed at the OrthoDB SPARQL endpoint webpage at https://sparql.orthodb.org. To execute Q8 and inspect its results, we provide the following link: http://purl.org/orthology/q8. Note that unlike OMA root HOGs where a gene can only be in one root HOG, in OrthoDB a gene can belong to multiple “root” HOGs. This is because OrthoDB might not explicitly relate the orthologous group built at the highest taxonomic level (i.e. the actual root) to lower level orthologous groups in the hierarchy. For example, for the HBB gene the Q8 query retrieves three distinct “root HOGs” with the following highest taxonomic levels: Eukaryota, Metazoa, and Vertebrata.

PREFIX orthodb: <http://purl.orthodb.org/>

PREFIX up: <http://purl.uniprot.org/core/>

SELECT DISTINCT ?hog_root ?species_name ?protein1_uniprot ?gene1 ?taxLevel_uniprot ?taxLevel

WHERE {

VALUES ?protein2_uniprot {<http://purl.uniprot.org/uniprot/P68871>}

?gene2 a orthodb:Gene.

?gene2 orthodb:memberOf ?groups.

?gene2 orthodb:memberOf ?hog_root.

FILTER NOT EXISTS {?hog_root orthodb:ancestralOG ?ancestor.}

?groups orthodb:ancestralOG* ?hog_root.

?gene1 a orthodb:Gene.

?gene1 orthodb:memberOf ?groups.

?gene1 up:organism ?organism.

?organism a ?taxon.

?taxon up:scientificName ?species_name.

?groups orthodb:ogBuiltAt ?taxLevel_uniprot.

?taxLevel_uniprot up:scientificName ?taxLevel.

?gene2 orthodb:xref ?xref2.

?xref2 orthodb:xrefResource ?protein2_uniprot.

?protein2_uniprot a orthodb:Uniprot.

?gene1 orthodb:xref ?xref.

?xref orthodb:xrefResource ?protein1_uniprot.

?protein1_uniprot a orthodb:Uniprot.

} ORDER BY ?hog_root, ?taxLevel

In further details, the Q8 query retrieves a set of 6-tuples (?hog_root ?species_name ?protein1_uniprot ?gene1 ?taxLevel_uniprot ?taxLevel ). The ?hog_root variable represents the “root HOG” that contains the gene represented with a given UniProt entry (e.g. P68871). The ?protein1_uniprot and ?gene1 variables are assigned the genes defined as UniProt and OrthoDB entries, respectively. These genes belong to the same root HOG (i.e. ?hog_root). In addition, ?species_name and ?taxLevel variables are assigned, respectively, the species scientific name and the taxonomic level (e.g. “Eukaryota”) where the gene is found. The ?taxLevel_uniprot is the corresponding UniProt URI of a ?taxLevel value.

### Protocol 4: Meta-analysis - comparing data across OMA and MBGD orthology

In this protocol, we show how to compare orthology information across multiple databases with SPARQL 1.1. Although the example in the following code fragment is restricted to OMA and MBGD, similar queries over different combinations of the orthology databases mentioned in this article can be derived based on the Code Fragments in Protocols 1, 2 and 3.

For a given UniProt entry such as the accession number K9Z723, retrieve orthologs that are **only** in MBGD, but not in OMA. Alternatively, to retrieve only those that appear in **both** sources, simply remove the "NOT" keyword in the FILTER clause below. To execute the query below denoted as Q9 at the OMA SPARQL endpoint, https://sparql.omabrowser.org/lode/sparql, we provide the following link: http://purl.org/orthology/q9

PREFIX oma: <http://omabrowser.org/ontology/oma#>

PREFIX orth: <http://purl.org/net/orth#>

PREFIX sio: <http://semanticscience.org/resource/>

PREFIX lscr: <http://purl.org/lscr#>

PREFIX mbgd: <http://purl.jp/bio/11/mbgd#>

SELECT ?protein2 ?species WHERE {

  SERVICE<http://sparql.nibb.ac.jp/sparql> {

    SELECT ?protein2 ?species ?uniprot_entry where {

                            VALUES ?uniprot_entry {<http://purl.uniprot.org/uniprot/K9Z723> }

                            ?cluster_mbgd a orth:OrthologsCluster.

                            ?cluster_mbgd orth:hasHomologous ?node1_mbgd.

                            ?cluster_mbgd orth:hasHomologous ?node2_mbgd.

                            ?node1_mbgd orth:hasHomologous* ?gene1.

                            ?node2_mbgd orth:hasHomologous* ?gene2.

                            ?gene1 mbgd:uniprot ?uniprot_entry.

                            ?gene2 mbgd:uniprot ?protein2.

                            ?gene2 mbgd:organism ?taxon.

                           OPTIONAL{?taxon mbgd:species ?species.}

                           FILTER(?node1_mbgd != ?node2_mbgd) } }

  FILTER NOT EXISTS { *# keep only those that do not exist in OMA*

              ?cluster a orth:OrthologsCluster.

              ?cluster orth:hasHomologousMember ?node1.

              ?cluster orth:hasHomologousMember ?node2.

              ?node1 orth:hasHomologousMember* ?protein_OMA_1.

              ?node2 orth:hasHomologousMember* ?protein_OMA_2.

              ?protein_OMA_1 lscr:xrefUniprot ?uniprot_entry.

              ?protein_OMA_2 lscr:xrefUniprot ?protein2.

              FILTER(?node1 != ?node2) }}

This federated SPARQL query will retrieve pairwise orthologous genes of the Cyanobacterium-aponinum psb27 gene that are found in the MBGD database but are not present in OMA. The psb27 gene is represented with its related UniProt entry, thus the UniProt URI http://purl.uniprot.org/uniprot/K9Z723.

### Aggregations in SPARQL: Combining data from multiple resources

By exploiting orthology databases that represent information with the same framework for data interchange (i.e. RDF) allow us to further query the data with the same query language (i.e. SPARQL). As a result, we can aggregate and combine data from multiple databases more efficiently. This is because we avoid the needs of syntactic conversions and changes in the original data models and structures by using SPARQL and RDF. These conversions and changes are often required by traditionals methods that combines different file-based data exchange formats or full database dumps. 

In the *Extended data,* we provide additional examples showing how to retrieve the top 10 entries with most orthologs in OMA and MBGD for a given species, e.g. *'Drosophila melanogaster'*. These examples illustrate a few more advanced SPARQL features, such as aggregation and ordering by a criterion in order to select the top N results.

## Conclusion

We provide four protocols that show how to query evolutionary relationships (pairwise orthologs, as well as HOGs) across four major databases available through SPARQL 1.1 endpoints: EBI, OMA, MBGD and OrthoDB. These protocols can serve as a useful starting point for readers interested in an introduction to the RDF data models of these data sources, as well as the basics of retrieving orthology information through SPARQL queries. Finally, we have shown how aggregations in SPARQL can be used to quickly generate an overview of the data available in each considered database, and how this data can be compared across the data sources.

To sum up, we hope these protocols provide a useful introduction into analysing evolutionary relationships among genes with SPARQL, as well as enriching these analyses by integrating information from external data sources, through federated queries. We have also integrated the queries in this article in the BioQuery search interface ( Sima *et al.,* 2019) available at http://biosoda.expasy.org/. Researchers to directly execute or further refine these queries in a more user-friendly environment.

We encourage readers to experiment with the example queries presented in this article, which are provided in the accompanying Jupyter notebook, to be directly re-used or integrated into existing research analysis pipelines. 

## Data availability

### Underlying data

Protocols (SPARQL queries) available from: https://github.com/biosoda/tutorial_orthology/blob/master/Orthology_SPARQL_Notebook.ipynb

Archived protocols as at time of publication: http://doi.org/10.5281/zenodo.3946639

License: CC0

### Extended data

Zenodo: Protocols to retrieve orthology information with SPARQL, http://doi.org/10.5281/zenodo.3946639 ( Sima & Mendes de Farias, 2020).

This project contains the following extended data:

- Table 1. "Cheat sheet" for RDF data available in the four sources considered in this tutorial. (*) GO annotations can be retrieved from the UniProt RDF store through UniProt cross-references.
- Supplementary protocols: Retrieving MBGD Homologous Groups
- Aggregation queries

Data are available under the terms of the Creative Commons Zero "No rights reserved" data waiver (CC0 1.0 Public domain dedication).

## Software availability

All the queries presented in the “Protocols” section are also now part of the BioQuery interface ( Sima *et al.,* 2019) available online at http://biosoda.expasy.org/. We included the queries in the “Information on Homologous Genes” section. Alternatively, they can be highlighted by searching for the database name, for example MBGD, in the search field on the top-left corner of the webpage. 

Furthermore, the accompanying Jupyter notebook allows for integration with other existing analysis tools by illustrating how to query SPARQL endpoints in Python, as well as how to export SPARQL query results as Pandas data frames.

### Online Data Access

SPARQL endpoints (all links include further example queries):

- ***OMA*** https://sparql.omabrowser.org/lode/sparql
- ***OrthoDB*** https://sparql.orthodb.org/
- ***EBI*** https://www.ebi.ac.uk/rdf/services/sparql
  - for example orthology queries see "Ensembl" category
- ***MBGD*** http://mbgd.genome.ad.jp/sparql/

## Footnotes

^1^ https://www.w3.org/TR/sparql11-query/#propertypaths
