# Supplementary material for: A hands-on introduction to querying evolutionary relationships across multiple data sources using SPARQL
Source: F1000Res. 2020 Jul 22;8:1822. Originally published 2019 Oct 29. [Version 2] doi: 10.12688/f1000research.21027.2 (PMC7324951; doi:10.12688/f1000research.21027.2)
Supplement: Peer review history

# Peer review: Reviewer response for version 2

**DOI:** 10.5256/f1000research.27892.r67708

**Published:** 2020-08-13

**Authors:** Ikuo Uchiyama

**Affiliations:** 1 Laboratory of Genome Informatics, National Institute for Basic Biology, National Institutes of Natural Sciences, Okazaki, Japan

I found that the manuscript has been carefully revised and I have no further comments.

Is the rationale for developing the new method (or application) clearly explained?

Yes

Is the description of the method technically sound?

Partly

Are the conclusions about the method and its performance adequately supported by the findings presented in the article?

Partly

If any results are presented, are all the source data underlying the results available to ensure full reproducibility?

Yes

Are sufficient details provided to allow replication of the method development and its use by others?

Partly

Reviewer Expertise:

Bioinformatics, specifically on comparative and evolutionary genomics and genome databases.

I confirm that I have read this submission and believe that I have an appropriate level of expertise to confirm that it is of an acceptable scientific standard.

# Peer review: Reviewer response for version 2

**DOI:** 10.5256/f1000research.27892.r67710

**Published:** 2020-08-12

**Authors:** Andra Waagmeester

**Affiliations:** 1 Micelio, Antwerp, Belgium

I am happy with the detailed responses of my earlier review. The issue with the availability of SPARQL results from one of the SPARQL endpoints remains. Still, the authors acknowledge that and provide solutions (i.e. contact details of support to the individual data providers. 

 "Given that copying, the data fragments to our Github repository might lead to several issues due to licensing problems, as well as to data size, we have made available a preview of the results directly in the Jupyter notebook."

 This is an interesting point, basically applicable licenses prevent proper reuse or reproducibility of research data, which should concern us. 

 All in all, I am pleased with the responses and additions and think it is an excellent paper that is a good example of how semantic web technologies can be applied in the life sciences.

Is the rationale for developing the new method (or application) clearly explained?

Yes

Is the description of the method technically sound?

Partly

Are the conclusions about the method and its performance adequately supported by the findings presented in the article?

Yes

If any results are presented, are all the source data underlying the results available to ensure full reproducibility?

Partly

Are sufficient details provided to allow replication of the method development and its use by others?

Partly

Reviewer Expertise:

Semantic web, Bioinformatics, data modelling, ontology development.

I confirm that I have read this submission and believe that I have an appropriate level of expertise to confirm that it is of an acceptable scientific standard.

# Peer review: Reviewer response for version 1

**DOI:** 10.5256/f1000research.23139.r64153

**Published:** 2020-06-29

**Authors:** Tatsuya Kushida

**Affiliations:** 1 National Bioscience Database Center, Tokyo, Japan; 2 RIKEN BioResource Research Center, Tsukuba, Japan

It can be useful to leverage orthologous gene knowledge in genome data analysis like gene function annotations, and the inference. The authors attempted to introduce how to retrieve the orthologous genes for several RDF databases such as OMA, MBGD, OrthoDB, and EnsemblRDF, using concrete SPARQL query examples. In particular, I highly evaluate that the authors explain through appropriate examples of the methods to retrieve the information of the complicated orthologous structures. It is expected to facilitate the understanding of the knowledge, and it would make contributions to increase the usage for genome data analysis. Moreover, the authors provided the Python codes' information to extract orthologous genes from the RDF databases and incorporate them into existing analysis pipelines. That is also worth appreciating. It is essential to further appeal the merits to bioinformaticians and experimental researchers in order to disseminate the orthologous RDF data.

 I realized that as stated in the section: Aggregations in SPARQL: Combining data from multiple resources (page 11), I can aggregate the data more efficiently using the SPARQL than traditional procedure (file-based data exchange or full database dumps.) On the other hand, the authors did not sufficiently mention the advantage of the RDF and SPARQL compared with the traditional procedure. I hope that the authors would more strongly emphasize the advantage.

Is the rationale for developing the new method (or application) clearly explained?

Yes

Is the description of the method technically sound?

Partly

Are the conclusions about the method and its performance adequately supported by the findings presented in the article?

Yes

If any results are presented, are all the source data underlying the results available to ensure full reproducibility?

Yes

Are sufficient details provided to allow replication of the method development and its use by others?

Partly

Reviewer Expertise:

Bioinformatics, Database integration, RDF, Ontology

I confirm that I have read this submission and believe that I have an appropriate level of expertise to confirm that it is of an acceptable scientific standard.

# Response

**Published:** 2020-07-08

**Authors:** Tarcísio Mendes

Dear Tatsuya Kushida,

 We would like to thank you for the insightful comments and suggestions for improvements. We have revised the article in order to take all comments into account. We thus hope that this new version takes into account the remarks and answers the questions raised.

 In summary, we have made the following changes:

- We have added all the queries presented in the article to the BioQuery3 interface online, (available at biosoda.expasy.org), Section "Information on Homologous Gene queries", making it easier for readers to try them out directly without requiring copy-pasting the SPARQL queries from the text of the article. Furthermore, we have also created clickable links that point users directly to the corresponding query results in the relevant SPARQL endpoint webpage, in the form of purl.org/orthology/q0 to purl.org/orthology/q9, where the ids Q0-Q9 correspond to the queries presented in the “Protocols” section.
- We have added a subsection "Applicable Ontologies", in the "Materials" section, explaining the ontologies relevant to the orthology domain, used by the four data sources described in the article (OMA, OrthoDB, MBGD and EBI).
- We have improved the explanations regarding the choice of relevant IRIs for formulating SPARQL queries targeting the orthology domain, while clarifying the specific challenges of querying orthology data with SPARQL.
- We have improved the OrthoDB data model figure with small corrections.
- We have included a subsection about “retrieving OrthoDB pairwise orthologs”. 

The following paragraphs list the issues identified by the reviewers, along with our corresponding answers. Below we provide detailed answers to your comments.

*It can be useful to leverage orthologous gene knowledge in genome data analysis like gene function annotations, and the inference. The authors attempted to introduce how to retrieve the orthologous genes for several RDF databases such as OMA, MBGD, OrthoDB, and EnsemblRDF, using concrete SPARQL query examples. In particular, I highly evaluate that the authors explain through appropriate examples of the methods to retrieve the information of the complicated orthologous structures. It is expected to facilitate the understanding of the knowledge, and it would make contributions to increase the usage for genome data analysis. Moreover, the authors provided the Python codes' information to extract orthologous genes from the RDF databases and incorporate them into existing analysis pipelines. That is also worth appreciating. It is essential to further appeal the merits to bioinformaticians and experimental researchers in order to disseminate the orthologous RDF data.*

- *I realized that as stated in the section: Aggregations in SPARQL: Combining data from multiple resources (page 11), I can aggregate the data more efficiently using the SPARQL than traditional procedure (file-based data exchange or full database dumps.) On the other hand, the authors did not sufficiently mention the advantage of the RDF and SPARQL compared with the traditional procedure. I hope that the authors would more strongly emphasize the advantage.*

**Authors: **We now included a better explanation to emphasize this advantage in the subsection “Aggregations in SPARQL: Combining data from multiple resources”.

# Peer review: Reviewer response for version 1

**DOI:** 10.5256/f1000research.23139.r55913

**Published:** 2020-06-12

**Authors:** Ikuo Uchiyama

**Affiliations:** 1 Laboratory of Genome Informatics, National Institute for Basic Biology, National Institutes of Natural Sciences, Okazaki, Japan

In this manuscript, the authors provide introductory examples showing how to query hierarchical orthology databases in RDF format using the SPARQL query language. Although these semantic web technologies are promising for integrating various biological resources, they still seem to be utilized by only very limited researchers. In this respect, this kind of introductory paper may be helpful for more biologists to utilize these technologies. However, for this purpose, I think that this manuscript has a serious defect that it is not easily understandable unless the readers have sufficient knowledge and skills of BOTH SPARQL and homology/orthology concepts (I do not think that this article contains any specific new ideas for such experts). Therefore, the authors should add some more explanation to improve the understandability for those who are not very familiar with either of these fields in order to achieve the goal of this article.

1. At the first example in "Data models" section (page 4), the authors should add some explanation about how to make a query based on a given RDF graph (with some figure). Such an explanation in addition to the RDF graphs of the actual databases can help understand the subsequent SPARQL codes.
2. Here, protocols are shown for extracting three types of homology/orthology information: pairwise orthologs, homologous groups, and hierarchical orthologous groups (HOGs), but the exact definitions of the extracted information are not clearly given. Moreover, the specifications of the protocols including their purposes and outcomes are not clearly explained. In fact, all of these protocols retrieve some kinds of pairwise homology/orthology relationships of a given UniProt entry, but the differences of them are not very obvious. Therefore, it is not easy for readers to understand these protocols exactly, and even impossible to check the correctness of the code, without any appropriate explanation. 
3. Related but somewhat minor comment: homologous group considered here seems to correspond to orthologous group that contains only orthologs and in-paralogs, while I think homologous group can generally also contain out-paralogs. 
4. Most readers are probably not satisfied with just a copy-and-paste of the given examples, but want to modify them for their own purposes, so it is desirable to give some hints on how to make modifications to each query.
5. Some codes containing double quotation marks did not work well when I did copy and paste them from the article web site, probably because these characters were converted into non-ASCII characters.
6. In the last example, the URIs of the same UniProt entry are appeared repeatedly. Can’t you use a common variable to specify the query URI only once?

Is the rationale for developing the new method (or application) clearly explained?

Yes

Is the description of the method technically sound?

Partly

Are the conclusions about the method and its performance adequately supported by the findings presented in the article?

Partly

If any results are presented, are all the source data underlying the results available to ensure full reproducibility?

Yes

Are sufficient details provided to allow replication of the method development and its use by others?

Partly

Reviewer Expertise:

Bioinformatics, specifically on comparative and evolutionary genomics and genome databases.

I confirm that I have read this submission and believe that I have an appropriate level of expertise to confirm that it is of an acceptable scientific standard, however I have significant reservations, as outlined above.

# Response

**Published:** 2020-07-08

**Authors:** Tarcísio Mendes

Dear Ikuo Uchiyama,

 We would like to thank you for the insightful comments and suggestions for improvements. We have revised the article in order to take all comments into account. We thus hope that this new version takes into account the remarks and answers the questions raised.

 In summary, we have made the following changes:

- We have added all the queries presented in the article to the BioQuery3 interface online, (available at biosoda.expasy.org), Section "Information on Homologous Gene queries", making it easier for readers to try them out directly without requiring copy-pasting the SPARQL queries from the text of the article. Furthermore, we have also created clickable links that point users directly to the corresponding query results in the relevant SPARQL endpoint webpage, in the form of purl.org/orthology/q0 to purl.org/orthology/q9, where the ids Q0-Q9 correspond to the queries presented in the “Protocols” section.
- We have added a subsection "Applicable Ontologies", in the "Materials" section, explaining the ontologies relevant to the orthology domain, used by the four data sources described in the article (OMA, OrthoDB, MBGD and EBI).
- We have improved the explanations regarding the choice of relevant IRIs for formulating SPARQL queries targeting the orthology domain, while clarifying the specific challenges of querying orthology data with SPARQL.
- We have improved the OrthoDB data model figure with small corrections.
- We have included a subsection about “retrieving OrthoDB pairwise orthologs”. 

The following paragraphs list the issues identified by you, along with our corresponding answers. Below we provide detailed answers to your comments.

*In this manuscript, the authors provide introductory examples showing how to query hierarchical orthology databases in RDF format using the SPARQL query language. Although these semantic web technologies are promising for integrating various biological resources, they still seem to be utilized by only very limited researchers. In this respect, this kind of introductory paper may be helpful for more biologists to utilize these technologies.*

- * However, for this purpose, I think that this manuscript has a serious defect that it is not easily understandable unless the readers have sufficient knowledge and skills of BOTH SPARQL and homology/orthology concepts (I do not think that this article contains any specific new ideas for such experts). Therefore, the authors should add some more explanation to improve the understandability for those who are not very familiar with either of these fields in order to achieve the goal of this article.*

    
**Authors: **To improve understandability we have added better explanations and citations throughout this article (see further details in the answers below).

- *At the first example in "Data models" section (page 4), the authors should add some explanation about how to make a query based on a given RDF graph (with some figure). Such an explanation in addition to the RDF graphs of the actual databases can help understand the subsequent SPARQL codes.*

**Authors: **We have added a new figure in the beginning of the “Data Models” section as well as an explanation regarding how to write a basic SPARQL query starting from this (simplified) graph model. We now also refer readers to an introductory book chapter we recently published on the topic.

  

- *Here, protocols are shown for extracting three types of homology/orthology information: pairwise orthologs, homologous groups, and hierarchical orthologous groups (HOGs), but the exact definitions of the extracted information are not clearly given. Moreover, the specifications of the protocols including their purposes and outcomes are not clearly explained. In fact, all of these protocols retrieve some kinds of pairwise homology/orthology relationships of a given UniProt entry, but the differences of them are not very obvious. Therefore, it is not easy for readers to understand these protocols exactly, and even impossible to check the correctness of the code, without any appropriate explanation. *

**Authors: **We agree that the terminology is confusing. In fact, in the meantime, we have published a separate F1000 paper which introduces the various types of orthologs used in OMA (https://f1000research.com/articles/9-27). We now refer to this, and have also added better explanations of the retrieved results for each query type (e.g. pairwise orthologs, homologous groups, and HOGs). To check the correctness of the retrieved results, the reader has to rely on potentially more time consuming alternative solutions provided by each independent database (e.g. RESTful APIs, Python libraries) or perform interactive searches on the omabrowser.org website.

  

- *Related but somewhat minor comment: homologous group considered here seems to correspond to orthologous group that contains only orthologs and in-paralogs, while I think homologous group can generally also contain out-paralogs. *

**Authors: **The reviewer is correct. We have added a sentence of explanation (“However, the kind of homologous groups considered in this tutorial do not contain “out-paralogs”—i.e. paralogs which result from gene duplications which took place prior to the last common ancestor of all species in the databases”).

  

- *Most readers are probably not satisfied with just a copy-and-paste of the given examples, but want to modify them for their own purposes, so it is desirable to give some hints on how to make modifications to each query.*

**Authors: **We have added an introduction on how to formulate a SPARQL query starting from an example data model graph, also pointing to the Ontology Lookup Service for mapping terms to their RDF identifiers (IRIs). We also indicate to the reader how to formulate SPARQL queries starting from the first data model introduced, MBGD, by following the direction of the arrows in the simplified data model figure. Finally, we have also added a dedicated subsection "Choosing the relevant target gene identifiers in RDF" explaining how to change the examples provided with the corresponding target genes of interest. Changing the queries should be facilitated by the availability of direct clickable links to the queries (see explanation below).

  

- *Some codes containing double quotation marks did not work well when I did copy and paste them from the article web site, probably because these characters were converted into non-ASCII characters.*

**Authors: **To solve this problem, we have now created clickable links that point users directly to the corresponding query results in the relevant SPARQL endpoint webpage. This avoids potential errors resulting from copy-pasting the formatted text into the relevant SPARQL endpoint webpage where the query is executed or possibly further modified.

 Furthermore, we have added all the queries presented in this article also to the BioQuery3 interface online, (available at biosoda.expasy.org), in the Section "Information on Homologous Gene queries", making it easier for readers to try them out directly without requiring copy-pasting the SPARQL queries from the text of the article. 

- * In the last example, the URIs of the same UniProt entry are appeared repeatedly. Can’t you use a common variable to specify the query URI only once?*

**Authors: **We have now replaced the repeated entry with a variable ?uniprot_entry. We also updated the Jupyter notebook accordingly.

# Peer review: Reviewer response for version 1

**DOI:** 10.5256/f1000research.23139.r55910

**Published:** 2019-11-27

**Authors:** Andra Waagmeester

**Affiliations:** 1 Micelio, Antwerp, Belgium

The authors describe how RDF and SPARQL can be instrumental in combining various resources with knowledge on homologs. The authors take the readers by the hand through some well thought examples of SPARQL queries. They have selected four resources. OMA, OrthoDB, EBI and MBGD. For each resource SPARQL queries are given.

 The paper is well written and a welcome contribution to the field. However, there are some issues:

 The paper could be improved with a section on applicable ontologies. This maybe as a subsection under materials, where the authors list the resources. Similarly, the used underlying ontologies/controlled vocabularies could be addressed. They are implicitly described in the data model sections, but since this is paper is also presented as a guideline on how to apply SPARQL, it would help the reader to understand how IRIs which are core to the semantic web are selected. Maybe also describe which ontologies in general apply to the narrative of this paper. See Malone *et al* ^1^.

 On various places in the paper the authors argue that SPARQL has a steeping learning curve. I disagree, SPARQL is not different from any other query language. I often argue the contrary, i.e. RDF and SPARQL are rather simple to learn. It is nothing more than wrapping your data in triples and select the proper iri's to represent the knowledge. It can't get any simpler. However, identifying the applicable IRIs and finding a balance between creating IRIs and reusing IRIs makes the process difficult.

 "Software availability". I would not call a SPARQL endpoint "software". I suggests that the mentioned SPARQL endpoints can be downloaded and installed locally. Maybe change to "online data access"?

 The paper would benefit from a software section, though. If possible it would be nice to show some tool integration. Similar to work by Putman T. *et.al.* ^2^ where linked data (through Wikidata) is rendered in a webapp.

 "If any results are presented, are all the source data underlying the results available to ensure full reproducibility?"

 I answered partly, because the SPARQL example queries do not always get results. The SPARQL query ran on the SPARQL endpoint of the EBI did not get results (The query under "a) . *Retrieving EBI pairwise orthologs").* Would it be possible to add captions and referencable numbers to the queries. The authors suggest to copy and paste the query to the applicable sparql endpoint. This might lead to coding issues, due different keyboard layouts that exist, worldwide. I would recommend using clickable links that lead to either a query (e.g. https://w.wiki/CrW) or its results (e.g. https://w.wiki/CrX).

 To deal with this (temporarily?) unavailability of the SPARQL endpoint, I would recommend storing the underlying data as rdf in the github repository which also contains the jupyter notebook mentioned. Or at least a subset big enough to demonstrate the results.

 It is than possible to reproduce the results using for example the following python gist

 ======

 from rdflib import Graph

 localGraph = Graph()

 localGraph.parse(

 query = """ SELECT * WHERE { ....

                   ....}

              """

 localGraph.query(query)

 etc

 ======

Is the rationale for developing the new method (or application) clearly explained?

Yes

Is the description of the method technically sound?

Partly

Are the conclusions about the method and its performance adequately supported by the findings presented in the article?

Yes

If any results are presented, are all the source data underlying the results available to ensure full reproducibility?

Partly

Are sufficient details provided to allow replication of the method development and its use by others?

Partly

Reviewer Expertise:

Semantic web, Bioinformatics, data modelling, ontology development.

I confirm that I have read this submission and believe that I have an appropriate level of expertise to confirm that it is of an acceptable scientific standard, however I have significant reservations, as outlined above.

## References

[1] : Ten Simple Rules for Selecting a Bio-ontology. *PLOS Computational Biology*.2016;12(2) : 10.1371/journal.pcbi.1004743 10.1371/journal.pcbi.1004743 PMC475099126867217

[2] : ChlamBase: a curated model organism database for the Chlamydia research community. *Database*.2019;2019: 10.1093/database/baz091 10.1093/database/baz091 PMC646344830985891

# Response

**Published:** 2020-07-08

**Authors:** Tarcísio Mendes

Dear Andra Waagmeester,

 We would like to thank you for the insightful comments and suggestions for improvements. We have revised the article in order to take all comments into account. We thus hope that this new version takes into account the remarks and answers the questions raised.

 In summary, we have made the following changes:

- We have added all the queries presented in the article to the BioQuery3 interface online, (available at biosoda.expasy.org), Section "Information on Homologous Gene queries", making it easier for readers to try them out directly without requiring copy-pasting the SPARQL queries from the text of the article. Furthermore, we have also created clickable links that point users directly to the corresponding query results in the relevant SPARQL endpoint webpage, in the form of purl.org/orthology/q0 to purl.org/orthology/q9, where the ids Q0-Q9 correspond to the queries presented in the “Protocols” section.
- We have added a subsection "Applicable Ontologies", in the "Materials" section, explaining the ontologies relevant to the orthology domain, used by the four data sources described in the article (OMA, OrthoDB, MBGD and EBI).
- We have improved the explanations regarding the choice of relevant IRIs for formulating SPARQL queries targeting the orthology domain, while clarifying the specific challenges of querying orthology data with SPARQL.
- We have improved the OrthoDB data model figure with small corrections.
- We have included a subsection about “retrieving OrthoDB pairwise orthologs”. 

The following paragraphs list the issues identified by you, along with our corresponding answers. Below we provide detailed answers to your comments.

*The authors describe how RDF and SPARQL can be instrumental in combining various resources with knowledge on homologs. The authors take the readers by the hand through some well thought examples of SPARQL queries. They have selected four resources. OMA, OrthoDB, EBI and MBGD. For each resource SPARQL queries are given.*

*The paper is well written and a welcome contribution to the field. However, there are some issues:*

- *The paper could be improved with a section on applicable ontologies. This maybe as a subsection under materials, where the authors list the resources. Similarly, the used underlying ontologies/controlled vocabularies could be addressed. They are implicitly described in the data model sections, but since this is paper is also presented as a guideline on how to apply SPARQL, it would help the reader to understand how IRIs which are core to the semantic web are selected. Maybe also describe which ontologies in general apply to the narrative of this paper. See Malone et al 1.*

**Authors**: We have added the subsection "Applicable Ontologies" in the "Materials" section. We explained the ontologies relevant to the orthology domain used by the four data sources described in the article (OMA, OrthoDB, MBGD and EBI). In particular, we described the Orthology Ontology (ORTH), but also several other controlled vocabularies used across the four data sources, such as GO annotations, the NCBI taxonomy etc.

     

- *On various places in the paper the authors argue that SPARQL has a steeping learning curve. I disagree, SPARQL is not different from any other query language. I often argue the contrary, i.e. RDF and SPARQL are rather simple to learn. It is nothing more than wrapping your data in triples and select the proper iri's to represent the knowledge. It can't get any simpler. However, identifying the applicable IRIs and finding a balance between creating IRIs and reusing IRIs makes the process difficult.*

**Authors**: We have rephrased the abstract to better point out the specific challenges of querying evolutionary data with SPARQL, in particular, formulating property paths to traverse the hierarchical orthologous groups (HOGs). We have also added the new subsections "Applicable Ontologies" and "Choosing the relevant target gene identifiers in RDF" with improved explanations  to identify possible relevant IRIs (for example, based on the applied vocabularies) to compose SPARQL queries in the context of the orthology domain. These queries use UniProt accession numbers as a starting point, and use cross-references available in each of the four data sources (OMA, OrthoDB, MBGD and EBI), to retrieve relevant data for target genes of interest. 

- *"Software availability". I would not call a SPARQL endpoint "software". I suggests that the mentioned SPARQL endpoints can be downloaded and installed locally. Maybe change to "online data access"? The paper would benefit from a software section, though. If possible it would be nice to show some tool integration. Similar to work by Putman T. et.al. 2 where linked data (through Wikidata) is rendered in a webapp.*

**Authors**: We have renamed the previous "Software availability" section on SPARQL endpoints to “Online Data Access” as suggested. We also added an entirely new section called "Software and Online Data Access", where we describe the integration of the queries in the BioQuery interface available online at biosoda.expasy.org.  

- *"If any results are presented, are all the source data underlying the results available to ensure full reproducibility?"I answered partly, because the SPARQL example queries do not always get results. The SPARQL query ran on the SPARQL endpoint of the EBI did not get results (The query under "a) . Retrieving EBI pairwise orthologs"). Would it be possible to add captions and referencable numbers to the queries. The authors suggest to copy and paste the query to the applicable sparql endpoint. This might lead to coding issues, due different keyboard layouts that exist, worldwide. I would recommend using clickable links that lead to either a query (e.g. https://w.wiki/CrW) or its results (e.g. https://w.wiki/CrX).*

**Authors**: We have created clickable links accordingly. These links point readers directly to the corresponding query results in the relevant SPARQL endpoint webpage. The links are in the form of purl.org/orthology/q0 to purl.org/orthology/q9 where the suffixes q0-q9 after the namespace “ purl.org/orthology/” correspond to the Q0-Q9 queries presented in the “Protocols” section (and are clearly marked in the article as such).

  

- *To deal with this (temporarily?) unavailability of the SPARQL endpoint, I would recommend storing the underlying data as rdf in the github repository which also contains the jupyter notebook mentioned. Or at least a subset big enough to demonstrate the results.*

**Authors**: Given that copying the data fragments to our github repository might lead to several issues due to licensing problems, as well as to data size, we have made available a preview of the results directly in the Jupyter notebook. We have included this explanation in the article. We have also added a clarification regarding the availability of the SPARQL endpoints and the relevant support addresses to contact (the support provided by each database owner) in case of long unavailability.
